# Identification of priority targets for intervention in outpatient antimicrobial stewardship

**DOI:** 10.1017/ash.2022.277

**Published:** 2022-08-05

**Authors:** Kellie N. Arensman Hannan, Evan W. Draper, Karen A. Uecker-Bezdicek, Eric O. Gomez-Urena, Kelsey L. Jensen

**Affiliations:** 1 Department of Pharmacy Services, Mayo Clinic Health System, Mankato, Minnesota; 2 Department of Pharmacy Services, Mayo Clinic, Rochester, Minnesota; 3 Division of Family Medicine, Mayo Clinic Health System, Fairmont, Minnesota; 4 Division of Infectious Diseases, Mayo Clinic Health System, Mankato, Minnesota; 5 Department of Pharmacy Services, Mayo Clinic Health System, Austin, Minnesota

## Abstract

A multimodal antimicrobial stewardship intervention was associated with a decrease in antibiotic prescribing for targeted non–coronavirus disease 2019 (COVID-19) upper respiratory infections from 27.6% in 2019 to 7.6% in 2021. We describe our approach to prioritizing departments for 3 levels of interventions in the setting of limited stewardship personnel.

An estimated 30% of outpatient antibiotic prescriptions in the United States are likely inappropriate, which has prompted the need for antimicrobial stewardship programs (ASPs) in the outpatient setting.^1^ As of 2020, institutions with ambulatory accreditation from The Joint Commission are required to implement an outpatient ASP under Standard MM.09.01.03. Components of this standard include the establishment of local leaders responsible for outpatient ASPs, selection of an annual goal related to antimicrobial use, adherence to evidence-based practices and guidelines pertaining to the annual goal, education of providers regarding ASP practices, and tracking and reporting of antimicrobial prescribing metrics.^2^

Acute upper respiratory infections (URIs) account for ∼44% of outpatient encounters in the United States that result in an antibiotic prescription, and only 50% of these prescriptions are likely appropriate.^1^ URIs, therefore, represent an optimal initial area of focus for new outpatient ASPs aiming to decrease inappropriate outpatient antibiotic prescribing.

The purpose of this study was to describe the implementation of a multimodal ASP intervention prioritized by potential for department-level improvement and its impact on antibiotic prescribing for targeted non–coronavirus disease 2019 (COVID-19) URI encounters within a variety of outpatient settings in a community health system.

## Methods

### Setting

The Mayo Clinic Health System–Southwest Minnesota comprises 5 hospitals with emergency departments and 13 outpatient clinics that include 3 urgent care departments, 2 pediatric and adolescent medicine departments, 1 community internal medicine department, and 11 family medicine departments. A single antimicrobial stewardship pharmacist is responsible for both inpatient and outpatient ASPs across the region.

### Data

An outpatient ASP dashboard within the institutional electronic health record (EHR) was created using Epic SlicerDicer (Epic, Verona, WI) to guide outpatient ASP efforts across the Mayo Clinic Enterprise.^3^ Diagnosis data were derived from *International Classification of Disease, Tenth Revision* (ICD-10) codes entered during visit-based encounters. URI ICD-10 codes were divided into diagnostic tiers using a previously validated schema based on whether antibiotics were always indicated (tier 1), sometimes indicated (tier 2), or not indicated (tier 3).^4^ The primary metric for the dashboard was antimicrobial prescribing rate for tier-3 diagnoses, calculated by dividing the total number of tier-3 visits in which antibiotics were prescribed by the total number of tier-3 visits for patients seen in visit-based encounters in the department specialties of family medicine, internal medicine, pediatrics, emergency medicine, and urgent care. ICD-10 codes for COVID-19 were not included in the denominator. Antimicrobial prescribing data can be viewed by region, clinic location, department specialty, and provider, and the data can be filtered by provider and patient demographics and by diagnosis.

### Intervention

The outpatient ASP goal for the Mayo Clinic Enterprise in 2021 was to reduce antibiotic prescribing in tier-3 URI encounters, with each region being responsible for implementing local initiatives to achieve a specific regional goal. Resources created for use across the enterprise available by January 2021 included a slide set for provider education, a patient-facing commitment poster, an EHR order panel with syndrome-specific recommendations, and symptomatic management guides for patients.^3^

Mayo Clinic Health System–Southwest Minnesota set a goal to reduce antibiotic prescribing for tier-3 URI indications from 27.6% in 2019 to <21.6% in 2021. Baseline data from 2019–2020 were reviewed to prioritize local outpatient ASP initiatives. Two urgent-care departments with a large number of tier-3 encounters (>300 per year) and a high rate of antibiotic prescribing (>40%) were identified as high-priority targets. All family medicine and pediatric and adolescent medicine departments were identified as moderate-priority targets due to moderate-to-high baseline prescribing rates (15%–57%) across these specialties but a lower number of tier-3 URI encounters (∼100–300 per year). The remaining urgent-care department, all emergency medicine departments, and all community internal medicine departments were identified as low-priority targets due to low baseline prescribing (<15%) and/or low numbers of tier-3 encounters (<100 per year).

At least 1 presentation was given to all departments between October 2020 and February 2021 to introduce the regional ASP goal and available resources. Communication techniques were discussed, and providers were encouraged to provide positive treatment recommendations for symptomatic management when not prescribing an antibiotic.^5^

**Fig. 1.**
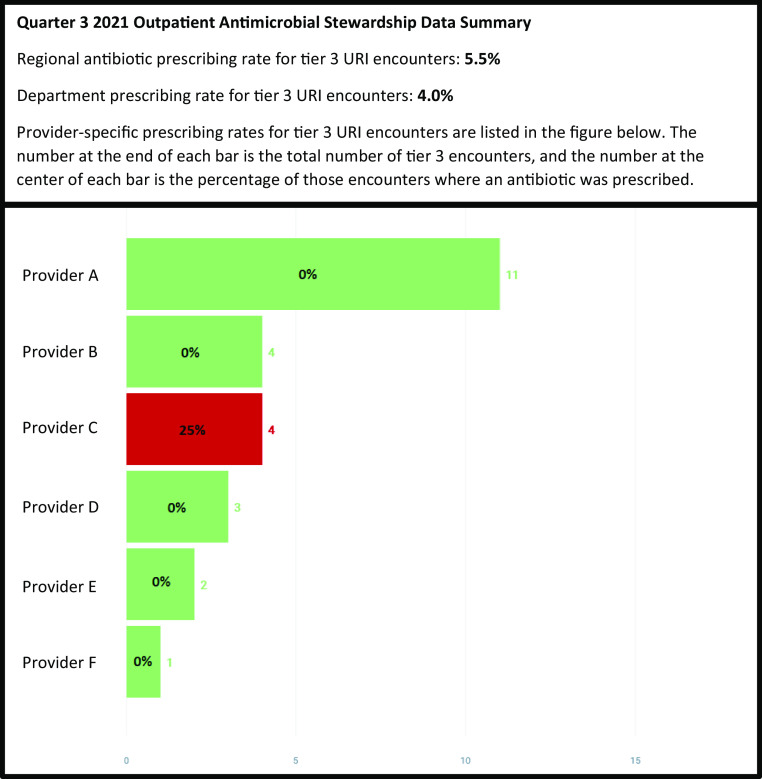
Example of a quarterly data summary send to a family medicine department.

In addition to receipt of departmental presentation(s), departments identified as moderate priority received quarterly data summaries in 2021 that included provider-specific antibiotic prescribing data (Fig. 1). For each of the high-priority departments, a provider ASP liaison was identified. These liaisons received additional one-on-one education from the ASP pharmacist and were sent monthly data for their departments. The ASP liaisons championed the initiative within their departments by encouraging use of the EHR order panel and symptomatic management guides and by reviewing antibiotic prescribing data with their peers.

This project was performed as a quality initiative and did not require review by the institutional review board.

## Results

Antibiotic prescribing during tier-3 URI encounters decreased from 27.6% in 2019 to 19.1% in 2020, with a further decrease to 7.6% in 2021 following the implementation of our intervention (Table 1). The 2019 data were included as a baseline due to the COVID-19 pandemic.

**Table 1.** Patient Characteristics and Antibiotic Prescribing for Tier-3 URI Encounters

Variable	2019	2020	2021
Patient age, median y (IQR)	21 (3–49)	24 (4–48)	16 (2–41)
Sex, female, no. (%)	6,860 (55.9)	4,241 (54.6)	3,891 (55.3)
Telemedicine or virtual visit, no. (%)	0 (0)	175 (2.3)	104 (1.5)
**All departments**			
No. of tier-3 URI encounters	12,274	7,771	7,036
Encounters with an antibiotic prescribed, no. (%)	3,384 (27.6)	1,488 (19.1)	536 (7.6)
**High-priority departments**			
No. of tier-3 URI encounters	1,840	1,051	1,285
Encounters with an antibiotic prescribed, no. (%)	870 (47.3)	446 (42.4)	103 (8.0)
**Moderate-priority departments**			
No. of tier-3 URI encounters	3,638	2,601	1,543
Encounters with an antibiotic prescribed, no. (%)	953 (26.2)	526 (20.2)	157 (10.2)
**Low-priority departments**			
No. of tier-3 URI encounters	6,796	4,119	4,208
Encounters with an antibiotic prescribed, no. (%)	1,561 (23.0)	516 (12.5)	276 (6.6)

Note. URI, upper respiratory infection; IQR, interquartile range.

## Discussion

We observed a decrease in antibiotic prescribing during outpatient tier-3 URI encounters in our community health system following implementation of a multimodal antimicrobial stewardship initiative. Chauhan et al^6^ successfully implemented a similar multimodal URI intervention within a geriatric clinic. We were able to implement our initiative on a larger scale by prioritizing departments to receive different levels of intervention.

We prioritized ASP interventions by department based on the volume of targeted encounters and baseline antibiotic prescribing rates. Previous studies have shown provider-level variability in antibiotic prescribing rates, supporting the use of peer-comparison data as an ASP strategy.^4^ We distributed provider-specific antibiotic prescribing data to moderate- and high-priority departments. Buehrle et al^7^ incorporated peer-comparison reporting within their multimodal outpatient ASP, and they observed a sustained decrease in antibiotic prescribing after peer-comparison reporting stopped. The creation of a provider-specific scoring tool for prioritizing ASP interventions directed toward individual providers may merit future investigation.

Antibiotic prescribing during targeted non–COVID-19 URI encounters began to decrease in 2020 prior to our ASP intervention. Ha et al^8^ noted a similar reduction in urgent-care antibiotic prescribing for non–COVID-19 respiratory conditions during the COVID-19 pandemic, particularly during telemedicine encounters. Although the reasons for this trend are unclear and likely multifactorial, this decrease in antibiotic prescribing is encouraging given prepandemic reports of high antibiotic prescribing rates during telemedicine URI encounters and associations with patient satisfaction scores.^9^

This study had several limitations. We used billing data to categorize URI encounters, and we did not evaluate the appropriateness of individual antibiotic prescriptions. We were not able to include prescriptions written outside encounter-based visits in our data model due to a lack required indications for antibiotic prescriptions in the outpatient setting. We did not apply statistical testing due to the retrospective, observational design of this study. Because we were not able to control for confounders such as the COVID-19 pandemic, our findings may be incidental and cannot be causally associated with the intervention.

Implementation of a multimodal ASP intervention was associated with a decrease in antibiotic prescribing during targeted non–COVID-19 outpatient URI encounters. Prioritization of specific departments or providers for ASP intervention based on rate of antibiotic prescribing and total number of encounters of interest may be advantageous in settings with limited ASP personnel.
